# Idiopathic hypereosinophilic syndrome in hemodialysis patients: Case reports

**DOI:** 10.1097/MD.0000000000025164

**Published:** 2021-03-12

**Authors:** Yuko Mutsuyoshi, Keiji Hirai, Junki Morino, Shohei Kaneko, Saori Minato, Katsunori Yanai, Hiroki Ishii, Momoko Matsuyama, Taisuke Kitano, Akinori Aomatsu, Haruhisa Miyazawa, Kiyonori Ito, Yuichiro Ueda, Susumu Ookawara, Yoshiyuki Morishita

**Affiliations:** Division of Nephrology, First Department of Integrated Medicine, Saitama Medical Center, Jichi Medical University, Saitama, Japan.

**Keywords:** corticosteroid therapy, hemodialysis, idiopathic hypereosinophilic syndrome

## Abstract

**Rationale:**

Herein, we report 3 hemodialysis patients with idiopathic hypereosinophilic syndrome who were successfully treated using corticosteroid therapy.

**Patient concerns:**

Case 1 was a 63-year-old man who was undergoing hemodialysis because of bilateral nephrectomy and developed hypereosinophilia with digestive symptoms, myocardial injury, and intradialytic hypotension. Case 2 was an 83-year-old man who was undergoing hemodialysis because of nephrosclerosis and developed hypereosinophilia with pruritus, myocardial injury, and intradialytic hypotension. Case 3 was a 59-year-old man who was undergoing hemodialysis because of diabetic nephropathy and developed hypereosinophilia with pruritus, myocardial injury, and intradialytic hypotension.

**Diagnoses:**

All 3 patients presented with hypereosinophilia (eosinophil count ≥1500 /μL for more than 1 month) and multiple-organ involvement (intradialytic hypotension, cardiac injury, digestive symptoms, and allergic dermatitis). A specific cause for the hypereosinophilia was not identified by systemic computed tomography, electrocardiography, echocardiography, bone marrow examination, or blood tests. Furthermore, Case 2 and 3 had not recently started taking any new drugs and drug-induced lymphocyte stimulation tests were negative in Case 1. Therefore, they were diagnosed with idiopathic hypereosinophilic syndrome.

**Interventions:**

All 3 patients received corticosteroid therapy with prednisolone at a dose of 40 mg/d, 30 mg/d, and 60 mg/d in Case 1, 2, and 3, respectively.

**Outcomes:**

Their digestive symptoms, pruritus, intradialytic hypotension, and serum troponin I concentrations were immediately improved alongside reductions in their eosinophil counts.

**Lessons:**

There have been few case reports of idiopathic hypereosinophilic syndrome in patients undergoing hemodialysis. We believe that recording of the clinical findings and treatments of such patients is mandatory to establish the optimal management of idiopathic hypereosinophilic syndrome.

## 1 Introduction

Idiopathic hypereosinophilia is a rare disease that is characterized by hypereosinophilia in the peripheral circulation and damage to multiple organs, including the lungs, heart, skin, nervous system, and gastrointestinal tract.^[1]^ It can cause irreversible organ dysfunction, disability, or death; therefore, urgent assessment and management are necessary. Symptoms and signs of hypereosinophilic syndrome vary a great deal, depending on the affected organs.^[1]^ In hemodialysis patients, hypereosinophilic syndrome has been reported to lead to hemodialysis intolerance, because of intradialytic hypotension or digestive symptoms.^[2,3]^ However, there have been few case reports of idiopathic hypereosinophilic syndrome in patients undergoing hemodialysis, and its clinical features remain to be fully characterized. Here, we report 3 cases of idiopathic hypereosinophilic syndrome in patients undergoing hemodialysis that were characterized by intradialytic hypotension and cardiac injury and were successfully treated using corticosteroid therapy.

## 2 Case report

**Figure 1:**
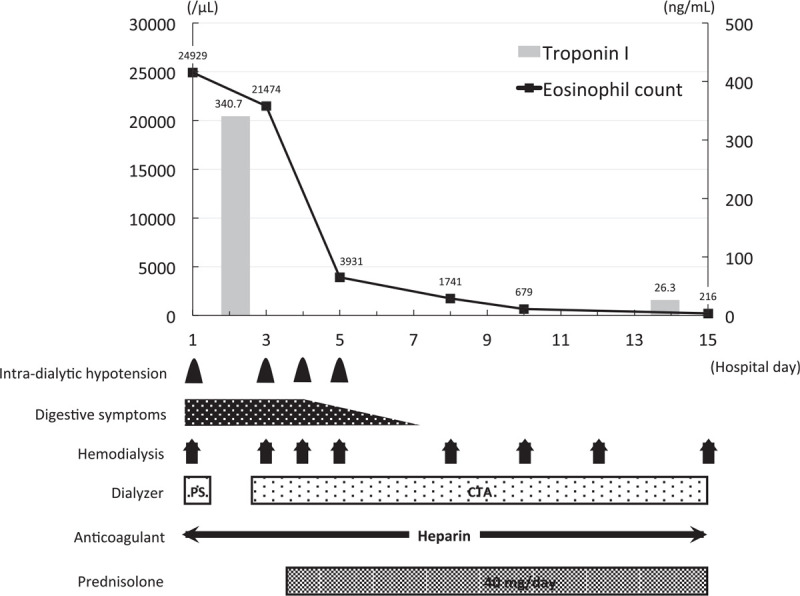
Clinical course of case 1. The *x* axis of the graph shows the number of days from admission and the *y* axis shows the eosinophil count (line) and the serum troponin I concentration (bars). The frequencies or durations of each clinical problem and treatment are shown below. CTA = cellulose triacetate, PS = polysulfone.

**Figure 2:**
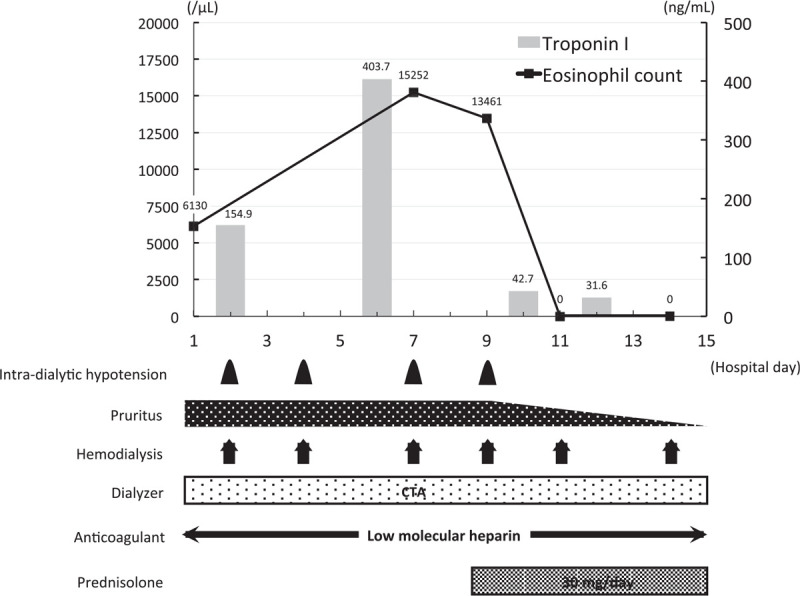
Clinical course of case 2. The *x* axis of the graph shows the number of days from admission and the *y* axis shows the eosinophil count (line) and the serum troponin I concentration (bars). The frequencies or durations of each clinical problem and treatment are shown below. CTA = cellulose triacetate.

**Figure 3:**
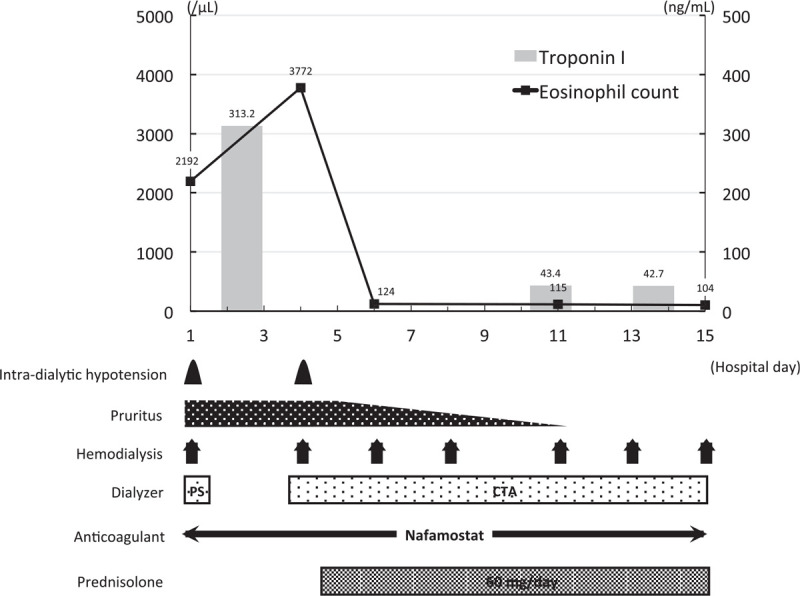
Clinical course of case 3. The *x* axis of the graph shows the number of days from admission and the *y* axis shows the eosinophil count (line) and the serum troponin I concentration (bars). The frequencies or durations of each clinical problem and treatment are shown below. CTA = cellulose triacetate, PS = polysulfone.

Table 1 summarizes the characteristics, dialysis prescriptions, treatments, and laboratory findings at the admission of the 3 patients. The treatment courses of each patient are illustrated in Figures 1–3.

**Table 1:** Patient characteristics, dialysis prescription, treatment, and laboratory findings at admission.

	Case 1	Case 2	Case 3	Reference range
Age (yr)	63	83	59	
Sex	Male	Male	Male	
Cause of end-stage renal disease	Bilateral nephrectomy	Nephrosclerosis	Diabetic nephropathy	
Allergy	None	None	None	
Symptoms	Digestive symptoms, myocardial injury, and intradialytic hypotension	Pruritus, myocardial injury, and intradialytic hypotension	Pruritus, myocardial injury, and intradialytic hypotension	
Involved organs	Digestive tract and heart	Skin and heart	Skin and heart	
Dialysis prescription				
Frequency (times a week)	3	3	3	
Time (hr)	4	4	3	
Dry mass (kg)	76.3	57.0	96.0	
Blood flow (ml/min)	200	200	200	
Dialysate flow (ml/min)	500	500	500	
Dialyzer	Polysulfone dialyzer (APS-21EA; Asahi Kasei Co, Ltd)	Polysulfone dialyzer (NV-21S; Toray Medical Co, Ltd)	Polysulfone dialyzer (NV-15S; Toray Medical Co, Ltd)	
Anticoagulation	Heparin (600 U/h)	Low-molecular weight heparin (600 U/h)	Nafamostat (30 mg/h)	
Treatment	Prednisolone 40 mg/d (0.5 mg/kg/d)	Prednisolone 30 mg/d (0.5 mg/kg/d)	Prednisolone 60 mg/d (0.6 mg/kg/d)	
Blood test results				
White blood cell count (/μL)	31,240	15,520	7830	3900–9800
Band (%)	0.4	0.0	1.0	0–19
Segmented (%)	13.4	43.5	55.0	25–72
Eosinophil (%)	79.8	39.5	28.0	0–7
Basophil (%)	0.4	0.5	1.0	0–2
Lymphocyte (%)	4.4	10.0	8.0	19–48
Monocyte (%)	1.0	6.0	2.0	3–9
Eosinophil count (μL)	24,929	6130	2192	0–500
Hemoglobin (g/dL)	9.9	10.4	11.3	12.0–17.6
Platelet count (×10^4^/μL)	21.6	14.2	20.3	13.0–36.9
Blood urea nitrogen (mg/dL)	36	42	38	8–20
Creatinine (mg/dL)	10.96	10.15	6.25	0.65–1.07
Aspartate aminotransferase (IU/L)	11	12	13	13–30
Alanine aminotransferase (IU/L)	2	13	7	10–42
Lactate dehydrogenase (IU/L)	179	341	339	124–222
Alkaline phosphatase (IU/L)	322	280	168	106–322
C-reactive protein (mg/dL)	2.0	2.7	6.0	0–0.14
Creatinine kinase (IU/L)	98	51	30	59–248
Creatinine kinase-MB (IU/L)	8	211	1	0–25
Troponin I (pg/mL)	340.7	154.9	313.2	<26.2
IgG (mg/dL)	818	1841	1798	870–1700
IgA (mg/dL)	199	609	367	110–410
IgM (mg/dL)	52	145	48	33–190
IgE (mg/dL)	29	1100	700	<173
MPO-ANCA (U/mL)	<1.0	<1.0	<1.0	<1.0

ANCA = antineutrophil cytoplasmic antibody, Ig = immunoglobulin, MPO = myeloperoxidase.

### 2.1 Case 1

The patient was a 63-year-old man who had been undergoing hemodialysis for ∼1 year because he had undergone bilateral nephrectomy to treat renal cell cancer. He had an 8-year history of diabetes mellitus and had recently experienced myocardial infarction. He was taking aspirin 100 mg/d, clopidogrel 75 mg/d, esomeprazole 10 mg/d, carvedilol 5 mg/d, rosuvastatin 2.5 mg/d, temocapril 2 mg/d, and a human insulin analog 10 units/d. An acetate dialysate and a dialyzer with a polysulfone dialysis membrane were being used for his hemodialysis. His medications and dialyzer membrane had not been changed for 6 months, with the exception of the aspirin, clopidogrel, and esomeprazole. One month before admission, eosinophilia (32.0%, 2659/μL) was identified, but he did not have any symptoms. One day before admission, he presented with fever, vomiting, and diarrhea and on the day of admission, he underwent hemodialysis. His systolic/diastolic blood pressure was 166/80 mmHg and his body temperature was 39.6°C. Shortly after the initiation of hemodialysis, his blood pressure dropped to 80/51 mmHg and he vomited. Therefore, the hemodialysis was stopped and he was admitted to the department. Blood tests revealed marked eosinophilia (79.8%, 24,929 /μL), high serum troponin I concentration (340.7 pg/mL), and impaired renal function (serum creatinine: 10.96 mg/dL, blood urea nitrogen: 36 mg/dL). Serological tests were negative for myeloperoxidase-anti-neutrophil cytoplasmic antibody and antiparasite antibodies. Drug-induced lymphocyte stimulation tests of aspirin, clopidogrel, and esomeprazole were negative. Thoraco-abdominal computed tomography revealed gastric and duodenal wall thickening, but there were no abnormalities in the lung fields. No abnormalities, except for those caused by the previous myocardial infarction, were noted on electrocardiography and echocardiography. Bone marrow examination revealed an eosinophil count of 42%, with no increase in blast count.

On the basis of these findings, the patient was diagnosed with idiopathic hypereosinophilic syndrome, with involvement of the digestive tract and heart. His dialyzer membrane was changed from polysulfone to cellulose triacetate, but the intradialytic hypotension and gastrointestinal symptoms did not improve. Therefore, oral prednisolone was started at a dose of 40 mg (0.5 mg/kg) per day on the 4th day of hospitalization, and his intradialytic hypotension and gastrointestinal symptoms were immediately ameliorated. On the 15th day of hospitalization, his eosinophil count and serum troponin I concentration had decreased to 216 /μL and 26.3 pg/mL, respectively, and the patient was discharged.

### 2.2 Case 2

The patient was an 83-year-old man who had been undergoing hemodialysis since the age of 80, because of nephrosclerosis. He had a history of undergoing surgery for sigmoid and ascending colon cancer at the ages of 77 and 80, respectively. He was taking lanthanum carbonate 1500 mg/d, febuxostat 10 mg/d, epinastine 20 mg/d, nalfurafine 5.0 μg/day, and magnesium oxide 500 mg/d. Bicarbonate dialysate and a dialyzer with a polysulfone dialysis membrane were being used for hemodialysis. His medications and dialyzer membrane had not been changed for 12 months. Three months before admission, he had presented with generalized pruritus and had eosinophilia (18.5%, 1905 /μL). One day before admission, he presented with hypotension (systolic blood pressure of 60 mmHg) and dyspnea during his hemodialysis session. Therefore, he was referred to our department for further examination and treatment. On admission, his systolic/diastolic blood pressure was 107/74 mmHg and his body temperature was 36.7°C. A generalized pruritic skin rash was observed. Blood tests showed marked eosinophilia (39.5%, 6130 /μL), high serum troponin I concentration (154.9 pg/mL), and impaired renal function (serum creatinine: 10.15 mg/dL, blood urea nitrogen: 42 mg/dL). Serological tests were negative for myeloperoxidase-anti-neutrophil cytoplasmic antibody and antiparasite antibodies. No abnormalities were detected on thoraco-abdominal computed tomography. No findings other than mild left ventricular hypertrophy were noted on electrocardiography and echocardiography. Bone marrow examination revealed an eosinophil count of 27%, with no increase in the blast count.

On the basis of these findings, the patient was diagnosed with idiopathic hypereosinophilic syndrome, with involvement of the skin and heart. All his medications were stopped and his dialyzer membrane was changed from polysulfone to cellulose triacetate. However, his intradialytic hypotension and pruritus did not improve. Therefore, oral prednisolone was started at a dose of 30 mg (0.5 mg/kg) per day on the 9th day of hospitalization, and his intradialytic hypotension and pruritus were immediately ameliorated. On the 14th day of hospitalization, his eosinophil count and serum troponin I concentration had decreased to 0/μL and 31.6 pg/mL, respectively. He was discharged on the 27th day of hospitalization.

### 2.3 Case 3

The patient was a 59-year-old man who had been undergoing hemodialysis for ∼1 year because of diabetic nephropathy that had developed as a complication of type II diabetes mellitus. He was taking aspirin 100 mg/d, carvedilol 1.25 mg/d, calcitriol 0.75 μg/d, calcium carbonate 1000 mg/d, esomeprazole 10 mg/d, and furosemide 160 mg/d. Acetate dialysate and a dialyzer with a polysulfone dialysis membrane were being used for hemodialysis. His medications and dialyzer membrane had not been changed for 5 months. Two months before admission, he had developed generalized pruritus and eosinophilia (22.0%, 1592 /μL). On the day of admission, he visited the hospital to undergo hemodialysis, and shortly after its initiation, his blood pressure dropped to 59/31 mmHg. Therefore, the hemodialysis was stopped. On admission, his systolic/diastolic blood pressure was 182/96 mmHg, his body temperature was 37.6°C, and a generalized pruritic skin rash was observed. Blood tests revealed marked eosinophilia (28.0%, 2192/μL), high serum troponin I concentration (313.2 pg/mL), and impaired renal function (serum creatinine: 6.25 mg/dL, blood urea nitrogen: 38 mg/dL). Serological tests were negative for myeloperoxidase-anti-neutrophil cytoplasmic antibody and antiparasite antibodies. No abnormalities were detected on thoraco-abdominal computed tomography. No findings except for mild left ventricular hypertrophy were made during electrocardiography and echocardiography. Bone marrow examination revealed an eosinophil count of 39%, with no increase in blasts.

On the basis of these findings, the patient was diagnosed with idiopathic hypereosinophilic syndrome, with involvement of the skin and heart. The dialyzer membrane was changed from polysulfone to cellulose triacetate, but his intradialytic hypotension and pruritus did not improve. Therefore, oral prednisolone was started at a dose of 60 mg (0.6 mg/kg) per day on the 5th day of hospitalization, and the intradialytic hypotension and pruritus were immediately ameliorated. On the 15th day of hospitalization, his eosinophil count and serum troponin I concentration had decreased to 104 /μL and 42.7 pg/mL, respectively and the patient was discharged on the 16th day of hospitalization.

## 3 Discussion

Eosinophilia (eosinophil count ≥500/μL) is a common finding in patients undergoing hemodialysis. It has been reported that the prevalence of eosinophilia in hemodialysis patients is 13% to 52%.^[4,5]^ Dialyzer membrane materials have been considered to be the principal cause of eosinophilia in hemodialysis patients,^[6,7]^ and in the present cases, the dialyzer membrane was changed from polysulfone to cellulose triacetate. However, this did not ameliorate the eosinophilia. Idiopathic hypereosinophilic syndrome is a systemic disease during which the eosinophil count increases in the peripheral circulation, which results in multiple-organ damage. It is diagnosed by the identification of hypereosinophilia (eosinophil count ≥1500/μL for more than 1 month) with multiple organ involvement (2 or more organs) and the exclusion of secondary causes of eosinophilia, including malignancy, eosinophilic leukemia, eosinophilic granulomatosis with polyangiitis, parasite infection, and drug reaction.^[1]^ In the present cases, the patients presented with hypereosinophilia and multiple-organ involvement (intradialytic hypotension, cardiac injury, digestive symptoms, and allergic dermatitis). A specific cause for the hypereosinophilia was not identified using systemic computed tomography, electrocardiography, echocardiography, bone marrow examination, or blood tests. Furthermore, 2 of the patients had not recently started taking any new drugs and drug-induced lymphocyte stimulation tests were negative in the third patient. Therefore, the 3 patients were diagnosed with idiopathic hypereosinophilic syndrome.

All of these patients had intradialytic hypotension and myocardial injury. Intradialytic hypotension has previously been reported in hemodialysis patients with hypereosinophilic syndrome.^[2]^ This is the result of the large number of eosinophils being activated by contact with the dialyzer membrane, leading to degranulation and the release of various cytokines,^[8]^ which increase vascular permeability and dilate capillaries.^[9,10]^ Major basic protein is also released by the eosinophils, and this is cytotoxic and induces tissue damage, including to cardiac muscle.^[11]^ The intradialytic hypotension and serum troponin I concentrations of the present patients were ameliorated alongside reductions in their eosinophil counts. Intradialytic hypotension and myocardial injury might be common in hemodialysis patients with idiopathic hypereosinophilic syndrome, but the details of further cases should be recorded to more thoroughly characterize this idiopathic hypereosinophilic syndrome.

Corticosteroid is recommended as the first-line therapy for hypereosinophilic syndrome.^[12]^ A previous study showed that approximately 80% of patients with hypereosinophilic syndrome had a favorable outcome following this treatment, but delayed intervention could lead to irreversible organ damage, disability, or death.^[13]^ In the present cases, corticosteroid was promptly administered after the diagnosis of hypereosinophilic syndrome, and the patients improved, rather than experiencing irreversible organ damage or disability. Further study of additional cases of idiopathic hypereosinophilic syndrome in patients undergoing hemodialysis is required to determine the most appropriate corticosteroid dose.

In conclusion, we have described 3 patients who were undergoing hemodialysis and developed idiopathic hypereosinophilic syndrome with intradialytic hypotension and cardiac injury. They were successfully treated using a corticosteroid, and their intradialytic hypotension and serum troponin I concentrations were ameliorated alongside reductions in their eosinophil counts.

## Acknowledgments

We thank Mark Cleasby, PhD, from Edanz Group for editing a draft of this manuscript.

## Author contributions

**Conceptualization:** Junki Morino, Shohei Kaneko.

**Data curation:** Saori Minato, Katsunori Yanai, Hiroki Ishii.

**Investigation:** Momoko Matsuyama, Taisuke Kitano, Akinori Aomatsu, Haruhisa Miyazawa.

**Supervision:** Kiyonori Ito, Yuichiro Ueda.

**Writing – original draft:** Yuko Mutsuyoshi.

**Writing – review & editing:** Keiji Hirai, Susumu Ookawara, Yoshiyuki Morishita.
